# Comprehensive optimization of urinary exfoliated tumor cells tests in bladder cancer with a promising microfluidic platform

**DOI:** 10.1002/cam4.5481

**Published:** 2022-12-25

**Authors:** Fengbin Gao, Jie Wang, Yanlan Yu, Jing Yan, Guoqing Ding

**Affiliations:** ^1^ Department of Urology, Sir Run Run Shaw Hospital Zhejiang University School of Medicine Hangzhou China; ^2^ Holosensor Medical Ltd. Suzhou China

**Keywords:** bladder cancer, CK20, diagnosis, urinary exfoliated tumor cells

## Abstract

**Background:**

Enrichment of urinary exfoliated tumor cells (UETCs) is a noninvasive way of bladder cancer diagnosis, but the lack of specific capture and identification of tumor cells from the urine remains a limitation that impedes the development of liquid biopsy.

**Methods:**

The CytoBot^®^ 2000, a novel circulating cell isolation and enrichment platform, was used for UETCs isolation after comprehensive optimization. The commercial cell lines of bladder cancer were used in spiking assay for cell recovery test. The flow cytometry and immunofluorescent staining assays were performed for expression validation of capture target and identification markers. The performance of optimized platform was validated by 159 clinical samples and analyzed using receiver operator characteristic curve.

**Results:**

The chip that had a pore diameter of 15*20 μm could reduce the background residues while maintaining a higher cell recovery rate. We found that the cell capture ability of chip significantly improved after anti‐EpCam antibody encapsulation, but not with T4L6FM1. In identification system optimization, the spiking assay and validation of clinical sample showed that the performance of CK20 and DBC‐1 were better that pan‐CK in tumor cell identification, in addition, the staining quality is more legible with CK20.

**Conclusion:**

The optimized capture chip is more specific for UETCs isolation. CK20 and DBC‐1 are both sensitive biomarkers of UETCs in bladder cancer diagnosis. The performance of this optimized platform is excellent in clinical test that improves the accuracy of urine cell testing and provides a new alternative for the clinical application of BLCA liquid biopsy assessment.

## Graphical abstract

In this study we use our novel CytoBot device for detecting urinary exfoliated tumor cells (UETCs) in the urine of bladder cancer patients. CytoBot specifically captures UETCs based on cell size and epithelial cell surface markers, which is verified via fluorescence microscopy. We optimise multiple parameters, including size of sieve holes and capture antibodies, and assess the diagnostic efficacy of UETC detection by CytoBot for bladder cancer patients.

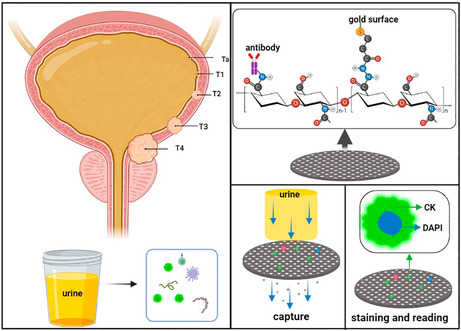

## 1 INTRODUCTION

Bladder cancer (BLCA, mostly urothelial type) is a malignant tumor that occurs in the mucosa of the urinary bladder^1^ and is the second most common cancer in the urinary system. It has the highest incidence of genitourinary diseases in China. According to GLOBOCAN (Global Cancer Observatory), in 2020, there was a total of 573,278 new cases of BLCA and 212,536 new deaths worldwide, and this number is anticipated to increase to 291,836 in 2030. Smoking and exposure to aromatic amine chemicals are considered as the two major risk factors in bladder tumorigenesis.^2^ For 30 years, however, clinicians and patients had access only to limited therapies. Most patients were diagnosed with advanced BLCA and 5‐year survival has remained flat.^3^

Urine cytology tests, ultrasound, and cystoscopy are the most common methods of diagnosis in BLCA.^4^ Treatment decisions are usually based on the pathological features of the primary tumor, so the necessary invasive biopsy is an indispensable part of the procedure depending on the location of the tumor. However, there are risks of complications and costs associated with tissue biopsy, which are worse when the lesion is located in a key organ or close to a primary blood vessel, making biopsy more challenging. In addition, cancer cells are known to continue evolving as adaptation to a changing microenvironment. Moreover, a single neoplastic tissue may contain several different molecular phenotypes of cells due to tumor heterogeneity,^5^ which considerably reduces the accuracy of cancer diagnosis and the outcomes of treatment.^6^ Currently, the most reliable method of cancer surveillance and treatment outcome assessment relies on radiography. However, this technique can only show tumor morphology at a certain time and place, and the utilization of radiological imaging and tissue biopsy in follow‐up significantly increases healthcare costs and reduces compliance and the life quality of patients.

Liquid biopsy is a non‐invasive biomarker test that holds promise in the diagnosis and monitoring of disease, and represents a major innovation in precision medicine.^7^, ^8^ Besides avoiding the disadvantage of limited longitudinal sampling, liquid biopsy is not subject to variation due to tumor heterogeneity.^9^ Blood, urine, saliva, and cerebrospinal fluid (CSF) are all available as liquid biopsies. Circulating tumor cells (CTCs), circulating nucleic acids (NAs), proteins, and exosomes isolated from fluids can be used as cancer biomarkers. In urological tumors, direct exfoliation of cancer cells into the urine, especially in carcinoma of the urethra, allows the use of the most appropriate and easily obtained medium for liquid biopsy.^10^ A variety of biomarkers in urine has been recommended since 2020 in the guidelines of the *National Comprehensive Cancer Network* (NCCN) and the *European Association of Urology* (EAU) for the diagnosis of urological cancers, including PCA3, PSA, NMP22, and others. Recently, a unique clinical trial of urine as a liquid biopsy use has been conducted (NCT04432909). This prospective multicenter and single‐blinded clinical trial aimed at determining the sensitivity and specificity of ultrasensitive chromosomal aneuploidy detection (UroCAD) analysis.^11^

However, the components of urine are very complicated. Urine samples include cells, free DNA of tumor cell origin, proteins, microvesicles, and metabolites. Compared to molecules such as NAs and proteins, the cytology test in urine provides a direct sign of tumorigenesis. Similar to pathology examinations, the cytology test of urine exfoliated tumor cells (UETCs) can be used to identify the pathological subtype of cancer and guide clinical treatment. Centrifugation and filtration are the most common methods for cell isolation from urine^12^ but are useless for enrichment of certain cells. ISET^®^, which collects circulating tumor cells in the blood with an 8‐μm filter but is often clogged by epithelial cells of larger size.^13^ The technique of targeted capture of tumor cells using immunomagnetic beads is widespread in blood‐based CTC detection, but with a high false‐positive rate in urine resulting from the lack of a specific biomarker, as in CellSearch^®^.^14^ Differently from blood, the composition of epithelial cells in urine is more complex, including urothelial cells, renal tubular epithelial cells, migratory epithelial cells, and squamous epithelial cells.^15^ These cells differ significantly in morphology, size, and expression of specific biomarkers, and efficient deletion of background/non‐targeted cells is the key to improving the accuracy of urine cytology tests and for the application of urine‐based liquid biopsy.

Here, we performed UETC capture with the help of a fully automated tumor cell isolation and enrichment platform, CytoBot^®^ 2000, produced by Holosensor Medical Ltd. Using this device, non‐target cells could be efficiently removed via holes in the chip; cancer cells are specifically intercepted by a high‐affinity chip and strongly bound by antibody coupled to the surface of the chip, followed by fully automated immunofluorescence staining and automated reading (the latter still pending) of the chip. The test result is available as rapidly as within only 3.5 h. With this highly reprogrammable system, we here propose improvements to the chip for the capture and identification of UETCs, and have verified the performance of this optimized platform in the present study.

## 2 MATERIAL AND METHODS

### 2.1 Clinical patients and urine samples

In this study, 89 BLCA patients were enrolled, with urine from 17 healthy volunteers as negative controls. All urine samples were collected, from March 2021 to December 2021 (Table 1). Patient tumor progression was confirmed by the clinician's diagnosis with urine testing, X‐ray, puncture, and cystoscopy, if necessary. Urine was collected before transurethral resection of bladder tumor (TURBT) and clinical treatment, medium‐range of urina sanguinis are required, 10 ml at least. Urine samples were sent to the laboratory within 6 h for UETC testing.

**TABLE 1:** Baseline of patients

Subject	*N*
Gender	
Male	60
Female	29
Age	
<50	7
≥50	82
Mean	66.22
Pathological type	
Follicular	1
Clear cell	1
Papillary	41
Squamous	1
Glandular	2
Grade	
Low	17
High	22
Tumor metastasis	
Positive	5
Negative	84

### 2.2 Urine pretreatment

On receipt of the urine samples, quality was first assessed, including hematuria, clarity, color, and volume. Ten milliliters of urine was collected after whirlpool blending. To remove impurities, urine was filtered through a mesh with a 40 μm diameter before centrifugation, 500 rpm for 10 mins, room temperature. Supernatant was removed and the cells were washed twice with PBS and finally adjusted to a constant volume of 300 μl.

### 2.3 Cell lines and culture

Bladder cancer cell lines 5637 (iCell‐h232, epithelial, from primary tissue of grade II carcinoma of BLCA) and T24 (iCell‐h208, epithelial, from transitional cell carcinoma) were purchased from iCell Bioscience Inc. Cells were cultured in RPMI‐1640 medium containing 10% fetal bovine serum (FBS) and 1% penicillin/streptomycin, and were incubated with 5% CO_2_, 37°C, at 70%–80% humidity.

### 2.4 Spiking assay

Tumor cells were digested by trypsin and washed twice with PBS, then resuspended. To model the condition of UETCs in urine, tumor cells were counted (50–60) and resuspended in the filtered and centrifuged urine, 10 ml, which was collected from patients who suffered from BLCA. This “mimic clinical sample” was pretreated as in previous steps (see “Urine pre‐treatment” above) and tumor cell isolation was performed by CytoBot^®^ 2000.

### 2.5 Enrichment of tumor cells and UETCs from the urine

Here, the tumor cells and UETCs were isolated using CytoBot^®^ 2000 (Holosensor Medical Ltd.). The procedure followed the manufacturer's protocol. In brief, the cell suspension was loaded onto the chip with a micropipettor, after which this fully automatic and smart device performed cell isolation and identification by itself. In this device, a micro‐pump drives the cell suspension over the capture chip at 50 μl/min. The tumor cells are captured by immunocapture through their expression of the epithelial cell surface biomarker, EpCam (Epithelial cell adhesion molecules). Next, tumor cell identification follows by immunofluorescence staining in situ. Antibodies to pan‐CK (ab215838, Abcam), CK20 (ab76123, Abcam) and DBC‐1 (YS‐M2761K, Yansheng) were selected for UETCs identification. CD45 (ab8216, Abcam) was used for leukocytes and DAPI (D9542, Sigma) for cell nuclear staining. After the program is finished, the chip is removed and the results read by immunofluorescence microscopy.

### 2.6 Flow cytometry

The digested cells were suspended in cell staining buffer (or PBS containing 1% BSA), centrifuged at 300 g for 5 min and the supernatant discarded. Washing was repeated once. Cells were resuspended at 1 × 10^7^/ml in an EP tube. Fc receptors were blocked (to reduce non‐specific staining during staining) for 10 min. Fluorescent antibody for staining was added according to the recommended dose in the provider's instructions and incubated for 30 min at 4°C, in the dark. Cells were then resuspended in staining buffer and centrifuged at 300 *g* for 5 min, resuspended in 0.2 ml staining buffer and finally analyzed by flow cytometry.

### 2.7 Immunofluorescence assay

The digested cells were fixed with 4% formaldehyde at room temperature for 15 min. and washed ×3 with PBS for 5 min each. Cells were blocked in blocking buffer for 60 min, after which the blocking solution was aspirated, primary antibody (AF‐488) was added and the cells were incubated at 4°C for 2 h. After washing ×3 in the dark, images were acquired.

### 2.8 Statistical analysis

All data in this study were analyzed by Graphpad Prism 7.0 (Graphpad software): unpaired *t*‐test, one‐way ANOVA, and Chi‐square testing were performed. For assessing the performance of tumor cell isolation by CytoBot^®^ 2000, ROC (Receiver Operating Characteristic) curves were generated by SPSS 24.0 (IBM). Data are shown as mean ± SEM, with a significant difference taken as *p* < 0.05.

## 3 RESULTS

### 3.1 Capture chip optimization for BLCA cell isolation

**FIGURE 1:**
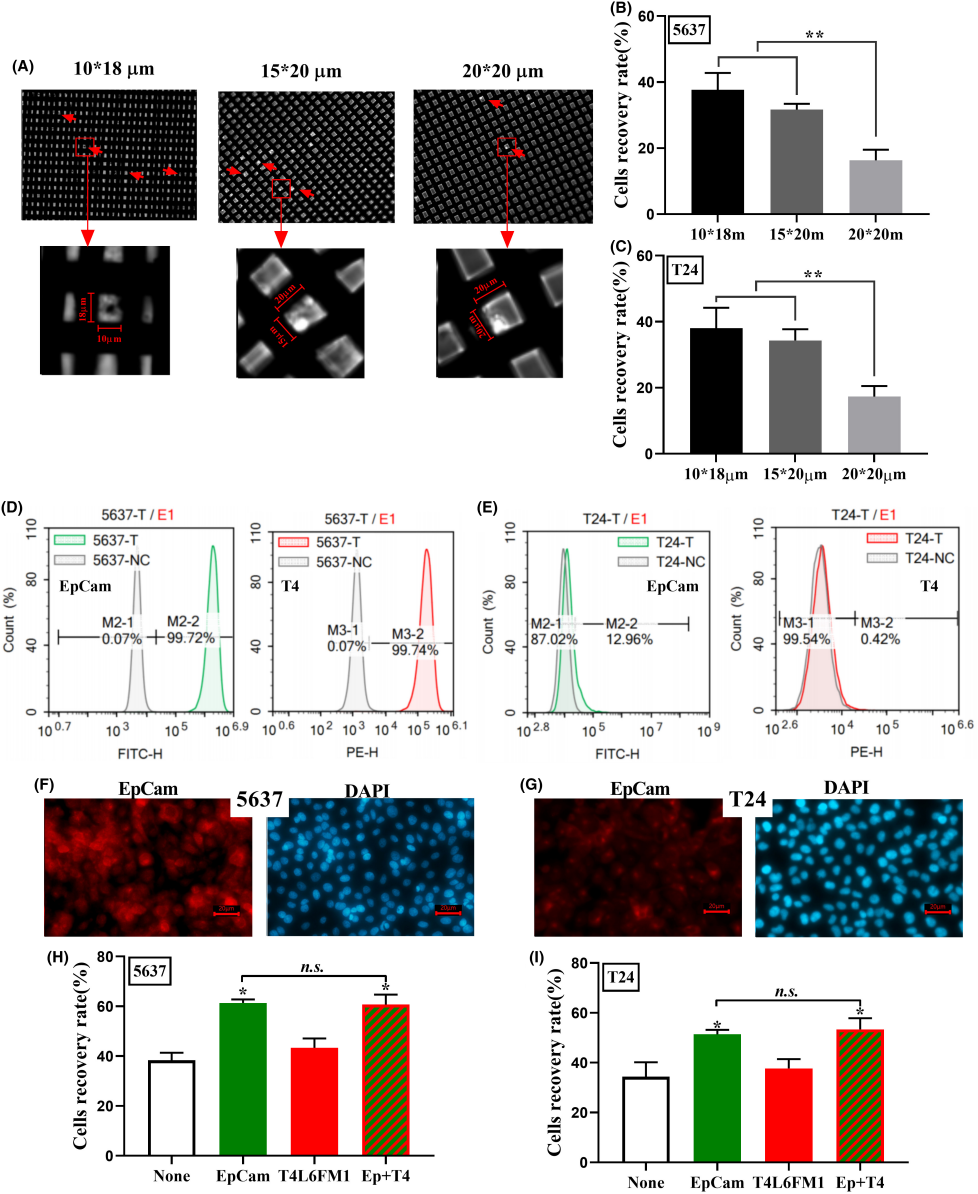
Optimizations of chip pore diameter and antibody encapsulation. (A) Images of chips and captured cells under bright field microscopy. Cells are indicated by red arrows. (B, C) Cell recovery rate of chips with different pore diameters, 5637 and T24 respectively. (D, E) Flow cytometry analysis of EpCam and T4L6FM1 expression on the surface of 5637 and T24 cells. (F, G) Immunofluorescence of EpCam in 5637 and T24 cells. Scale bar = 20 μm. (H, I) Cell recovery rate of chips with different antibody encapsulation in 5637 and T24 cells. Data are shown as mean + SEM, one‐way ANOVA, *p* > 0.05, *n.s*.; *p* < 0.05, *; *p* < 0.01, **.

Clearly quite different from blood cells, the composition of epithelial cells in urine is very diverse. For the purpose of capturing UETCs from urine with higher sensitivity, we first screened and optimized the CytoBot^®^ 2000 chip, specifically in terms of the pore size and entrapment by the capture antibody on the chip. Here, three different pore diameters were selected for tests of the cell recovery rate of BLCA cell lines (5637 and T24) without antibody. The pore diameters of the three metal‐prepared chips were 10*18 μm, 10*20 μm, and 20*20 μm (Figure 1A). The data show a lower recovery (<20%) of cells by chips with a pore diameter of 20*20 μm, and the smaller pore diameter of the chip shows a higher cell recovery rate. The cell recovery rate of the chip with a pore diameter 15*20 μm was slightly lower than that of the chip with the smallest pore diameter, but this difference was not significant, statistically (Figure 1B,C).

In order to obtain the target cells with as little interference as possible, we chose the chip with a pore diameter of 15*20 μm for the second step optimization, which was antibody selection for immunocapture, mainly with EpCam and T4L6FM1. EpCam is a membrane protein specifically expressed on epithelial cells and is widely used for CTC capture from the blood.^14^ T4L6FM1 which was recommended by Prof. Jerome (University of Warwick) is also a membrane protein expressed on epithelial cells, but little work has been reported on this biomarker. Here, we first examined the expression levels of EpCam and T4L6FM1 in BLCA cells using flow cytometry and immunofluorescence (IF), respectively. The results showed that 5637 cells express high levels of both EpCam and T4L6FM1 (Figure 1D), while the expression of these molecules was relatively low in T24 (Figure 1E). We only performed IF analysis for EpCam expression here, the IF of T4L6FM1 is unavailable because antibody application limitation. The results indicate a relatively higher fluorescence intensity of EpCam in 5637 (Figure 1F,G). These data suggest that the BLCA cell line 5636 is strongly positive for EpCam and T4L6FM1, while T24 is weakly positive. We then specifically conjugated the antibody to the chip with the help of HA (hyaluronic acid), which forms a flexible polymer layer on the surface of the metal chip that protects and arrests the target cells. There is an increased recovery rate of cancer cells when the chip is incubated with both of these antibodies. The data show that encapsulation of the EpCam antibody improved cell recovery most significantly (more than 60%), and that this coupled chip is more effective in recovering 5637 than T24 cells due to the strong EpCam positivity of the former (Figure 1H,I). The data also show that encapsulation of the T4L6FM1 antibody did not noticeably improve cell recovery either for 5637 or T24 (Figure 1H,I). However, no additive improvement was observed when both antibodies were enveloped (Figure 1H,I).

### 3.2 Identification optimization of UETCs

**FIGURE 2:**
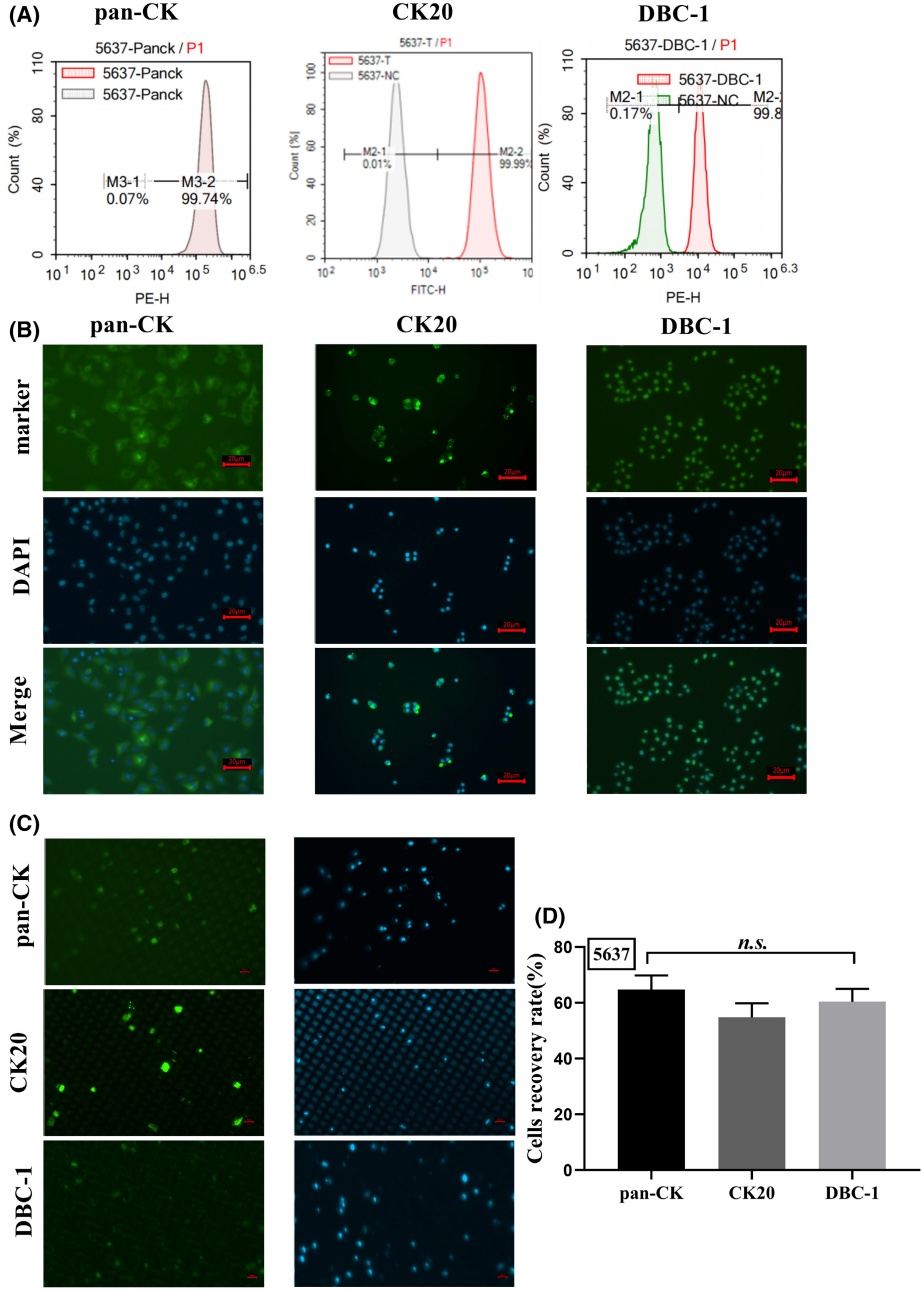
Verification of pan‐CK, CK20, and DBC‐1 identification in the 5637 cell line. (A) Flow cytometry analysis of expression of pan‐CK, CK20, and DBC‐1 in 5637. (B) Immunofluorescence of pan‐CK, CK20, and DBC‐1 in 5637. (C) Spiking assay for 5637 in urine with pan‐CK, CK20, and DBC‐1. (D) Cell recovery rate of 5637 cells identifying with pan‐CK, CK20, and DBC‐1. Data are shown as mean + SEM, one‐way ANOVA, *p* > 0.05, *n.s*. Scale bar = 20 μm.

**FIGURE 3:**
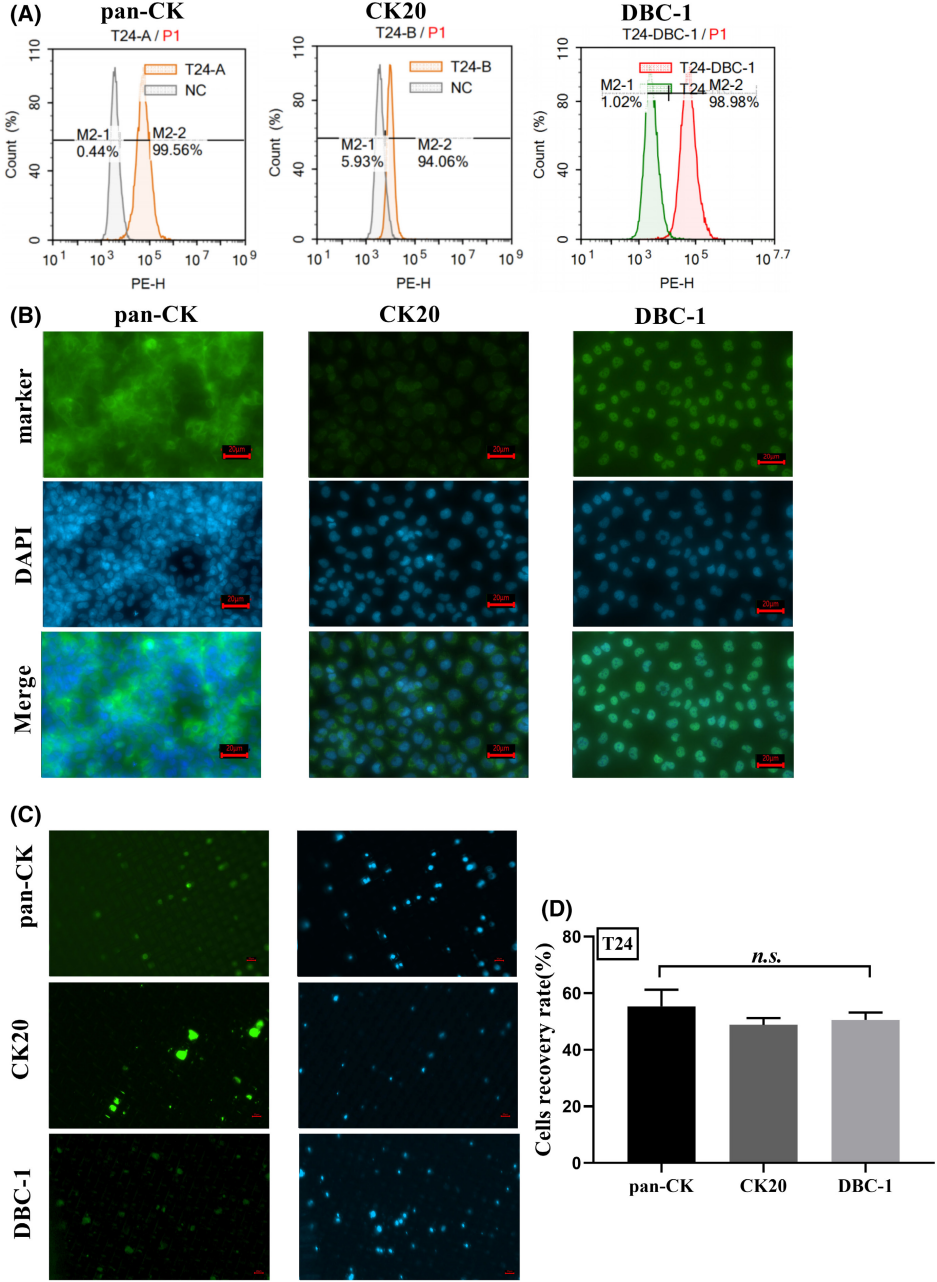
Verification of pan‐CK, CK20, and DBC‐1 identification in the T24 cell line. (A) Flow cytometry analysis of expression of pan‐CK, CK20, and DBC‐1 in T24. (B) Immunofluorescence of pan‐CK, CK20, and DBC‐1 in T24. (C) Spiking assay for T24 cells in urine with pan‐CK, CK20, and DBC‐1. (D) Cell recovery rate of T24 cells identifying with pan‐CK, CK20, and DBC‐1. Data are shown as mean + SEM, one‐way ANOVA, *p* > 0.05, *n.s*. Scale bar = 20 μm.

Pan‐CK is a protein molecule widely expressed in epithelial‐like cells and has a low specificity for tumor cells in urine. To improve the specificity and accuracy of cell detection in the urine of BLCA patients, we here validated the specificity of two other BLCA markers (CK20 and DBC‐1) with the aid of the CytoBot^®^ 2000 platform. First, we compared the expression of pan‐CK, CK20, and DBC‐1 by BLCA cells (5637 and T24) using flow cytometry and IF assays, respectively. The results showed that 5637 and T24 were both positive for pan‐CK, CK20, and DBC‐1 (Figure 2A,B, Figure 3A,B). As can be observed from the IF images, the fluorescence intensity of CK20 is stronger than pan‐CK and DBC‐1 in 5637 cells (Figure 2B), but is weak in T24 cells as shown in Figure 3B. In contrast, the intensity of DBC‐1 staining in both cell lines is more similar (Figure 2B, Figure 3B).

Further, a spiking assay was performed for marker validation of BLCA by CytoBot^®^ 2000 platform using cell‐free patient urine spiked with known numbers of cells from cancer cell lines. In brief, 60 cancer cells were spiked into 10 ml of patient urine (which had been filtered and centrifuged) and followed with centrifugation, washing, and resuspension and then performed cell isolation and staining with CytoBot^®^ 2000. EpCam antibody‐coated chips with a pore diameter of 15*20 μm were used for this test. The results showed that pan‐CK and CK20 were more effective in identifying BLCA cells and the images were easy to interpret, but the fluorescence of DBC‐1 was very weak and it was more difficult to identify and enumerate the cancer cells (Figure 2C and Figure 3C). We also found that CK20 had less non‐specific staining than pan‐CK (Figure 2C and Figure 3C). However, the results showed no significant difference between cell recovery rates when using pan‐CK, CK20, or DBC‐1, which were all around 60% (Figure 2D and Figure 3D).

To compare the specificity of different markers for UETC identification, we further performed IF assays with pan‐CK, CK20, and DBC‐1 in urine collected from healthy individuals, which were not spiked with cancer cells. Due to the low presence of cells in the urine of normal healthy people, 50 ml of urine was collected and cells were obtained after centrifugation, then stained with specific antibodies and DAPI on glass slides. The results showed that CK20 and DBC‐1 were both present at lower levels in normal urine cells relative to pan‐CK (Figure S1). This implies that there will be less false‐positive events in urine cell tests performed using CK20 and DBC‐1 antibodies.

### 3.3 Urine biopsy in BLCA patients by CytoBot ^®^ 2000 using different biomarkers

**FIGURE 4:**
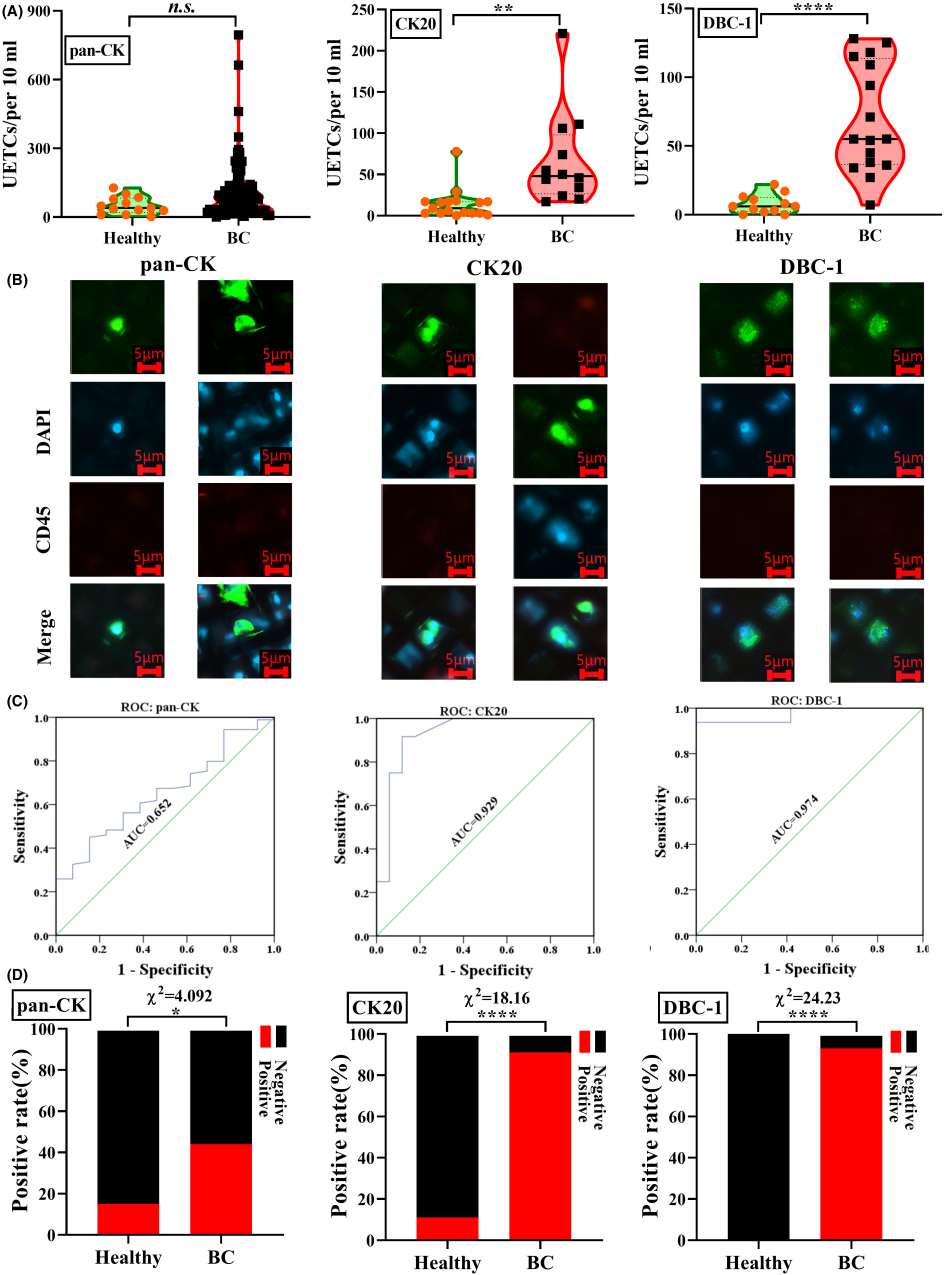
Clinical performance verification of pan‐CK, CK20, and DBC‐1 identification. (A) Clinical urine tests with pan‐CK, CK20, and DBC‐1, respectively. (B) Images of UETC identified by pan‐CK, CK20, and DBC‐1, respectively. Scale bar = 5 μm. (C). ROCs of pan‐CK, CK20, and DBC‐1 in clinical urine tests. (D) UETC positive rate in clinical urine tests identified by pan‐CK, CK20, and DBC‐1, respectively. Chi‐square, *p* < 0.05, *; *p* < 0.0001, ****.

To examine the efficacy of pan‐CK, CK20, and DBC‐1 for the identification of UETCs of BLCA patients, we next tested clinical samples. In combination with previous clinical tests, data of UETC tests obtained from 117 urine samples of BLCA patients (89, 11, and 16 with pan‐CK, CK20, or DBC‐1, respectively) and 42 controls (13, 17, and 12 with pan‐CK, CK20, or DBC‐1, respectively) were compared. We found no significant differences in the number of UETC between BLCA patients and healthy individuals when using pan‐CK as the indicator (107.8 > 50.8, *p* = 0.1140), while more UETCs were found in BLCA patients than in healthy controls when using CK20 or DBC‐1 as the indicator, that is 66.8 > 14.1 (*p* = 0.0014) and 69.4 > 7.7 (*p* < 0.0001), respectively (Figure 4A). However, in terms of staining quality, the cell morphology and clarity in Pan‐CK and CK20 identification systems were significantly better than DBC‐1 (Figure 4B). We then constructed ROC curves to assess the sensitivity and specificity of clinical urine tests with three indicators in BLCA. As shown in Figure 4C, the areas under the curve (AUC) were 0.652, 0.929, and 0.974 respectively, indicating that CK20 and DBC‐1 have excellent sensitivities and specificities for UETC identification in urine testing. We obtained cut‐off values (Youden = Sensitivity+Specificity‐1) of 86.5, 19, and 24.5 for pan‐CK, CK20, and DBC‐1, respectively, with sensitivities and specificities of 44.9% and 84.6%, 91.7% and 88.2%, 93.8% and 100% (Table S1–S3). Chi‐square testing was performed to assess the positive rate of UETC diagnosis in BLCA urine with pan‐CK, CK20, and DBC‐1 at each cut‐off value, the result indicating significant differences of differential diagnosis between the BLCA and healthy controls in all three UETC biomarkers. However, pan‐CK displayed the poorest result and positive rate. In contrast, CK20 and DBC‐1 both had excellent efficacy (>90%) of BLCA diagnosis (Figure 4D).

## 4 DISCUSSION

The urine cytology test is an integral part of the clinical screening, diagnosis, and long‐term surveillance of BLCA and plays an important role in the identification of clinicopathological type and localization of cancerous tissue. The most crucial issue of the urine cytology test is the specific capture of epithelial cells and the precise identification of cancer cells.^16^ In recent years, the popularity of CTC detection has led to the rapid growth of platforms for the isolation and enrichment of circulating cells. However, these devices are very poorly suited to clinical urine cell testing because of the high rate of false‐positive events. These are caused by the large number of cell fragments in urine which make the use of EpCam‐based immuno‐magnetic beads problematic.^17^ Physical enrichment with a mesh based on cell size can be used to overcome this problem, but results in the loss of target cells which reduces accuracy.^18^ In the present study, we assessed a highly reprogrammed platform for circulating cell isolation and enrichment, CytoBot^®^ 2000. In this device, target cells are isolated relying on a chip with a changeable pore diameter and enveloped antibodies, and are then recognized by a customizable identification system. Hence, we have optimized the chip and cancer cell identification system of the device specifically according to the characteristics of BLCA cells in urine. We show that the optimized chip is more specific for the capture of UETC, while there is an improved sensitivity (91%–94%) and specificity (88%–100%) in clinical urine tests with the new system that recognizes tumor cells on the basis of novel BLCA biomarkers. There are many recent reports on applications of similar cell enrichment devices with microchannels for urine cell separation, such as the MicroUPSD,^19^ which performs cell isolation also with an EpCam‐based microfluidic chip. While an extended microfluidic channel increases the chances of antibody‐cell engagement, it also simultaneously increases the time–cost and the challenges of target cell collection. However, using the chip introduced in the present study, the UETCs captured on the chip surface are easy to collect for further analysis. In addition, this capture program is accomplished in 10 min, which improved the test efficiency.

Advances in detection technology have driven a great increase in the number of molecular biomarkers for tumor detection. However, the specificity of these markers for certain cancer recognition is still insufficient. CK (cytokeratin) is a protein molecule widely expressed by epithelial‐like cells and is a very effective marker employed for the screening of blood CTCs due to the absence of epithelial cells in the blood under normal conditions.^20^ However, the presence of a large number of epithelial cells of different types in the urine leads to a reduction in the specificity of urine tumor cells identification. Therefore, a biomarker with high specificity and sensitivity is the key to improving the accuracy of liquid biopsy in BLCA, even in general clinical diagnosis. Here, we propose two specific markers for BLCA cell identification, CK20 and DBC‐1. CK20 is a member of the CK family and has been established as a protein marker that is significantly expressed in BLCA, making identification of the target cells more precise.^21^ The gene encoding DBC‐1 is located within a chromosomal region that shows loss of heterozygosity in some BLCAs. It contains a 5’ CpG island that may be a frequent target of hypermethylation, and it may undergo hypermethylation‐based silencing in some BLCAs (provided by RefSeq); hence, its specificity in BLCA needs further validation. Our results showed that relative to pan‐CK, the specificity (93%–100%) of CK20 and DBC‐1 are excellent for BLCA diagnosis by urine cell testing while the sensitivity of these two indicators also performs well in the validation of clinical urine samples. Simone et al.^21^ found that there is a significant correlation between cancer‐specific survival (CSS) and the presence of CK20+ cells, when combined with Ki67 staining. Karsten et al.^22^ strove to obtain a specific indicator for BLCA and reported that the diagnostic performance of CK20 transcript levels is similar to voided urine cytology. In addition to CK20, other emerging biomarkers are being claimed to be able to specifically label BLCA cells. NMP22 is the most popular target in clinical urine tests, and has a high specificity and sensitivity for the diagnosis of BLCA.^23^ However, positive results can also be obtained in some cases associated with benign urinary tract disease or prostate cancer.^24^ Due to the disappointing performance of single markers, multi‐marker panels are mostly used in clinical practice for BLCA diagnosis. A study recently reported that combining two tests targeting immunocytes, cytology, FISH, and NMP22, resulted in a sensitivity and specificity of 89.8% (immunocytes + NMP22) and 92.1% (FISH + immunocytes).^25^

As mentioned above, cells in the urine are so complex that their classification is difficult. The most commonly used approach is currently urine cytology tests, where cells are centrifuged, stained, and inspected to determine cancerous cells and morphology. Cystoscopy and tissue tests are then required for confirmation.^4^ Similarly, CellDetect also distinguishes malignant and benign tumor cells by pathological staining and cell morphology,^26^ based on differences in glycolytic metabolism between malignant and normal cells. At present, it seems that cytopathological staining is still the gold standard for urine cell detection. However, in the last decade, non‐radioactive chemiluminescence immunoassays have gained in acceptance because they are characterized by high sensitivity and specificity, straightforward analytical methodology, stable performance, and lower uncertainty. Thus, specific tumor markers are showing great potential in immunochemiluminescence assays. In the present study, many types of epithelial cells were identified by the specific antibody CK20 in urine cells (Figure 2A), and the morphology of these cells was clearly visible by fluorescence microscopy. Possibly, immunochemiluminescence will be useful in cell morphology investigations. In addition, the expression level of PD‐L1 is an important indicator for clinical treatment of BLCA,^27^ but this value cannot be obtained from conventional urine cytopathological tests. The CytoBot^®^ 2000 platform we used here also provides information about the expression of PD‐L1 on tumor cells (Figure 2B). This system greatly improves the efficiency of clinical diagnosis by obtaining information on urine cell morphology, expression of tumor markers, and drug targets simultaneously.

## 5 CONCLUSION

With the purpose of more efficient collection of UETCs from BLCA urine, the CytoBot^®^ 2000 platform was used and specifically optimized here. The most suitable chip parameters and new cancer cell recognition markers have been investigated in cellular experiments and fully validated by the spiking assay and clinical samples. The CytoBot^®^ 2000 is a promising clinical diagnostic tool for cell‐based tests, and CK20 and DBC‐1 were verified as more sensitive and specific markers of UETCs in urine cytology test of BLCA.

## AUTHOR CONTRIBUTIONS

Fengbin Gao: Analysis and interpretation of data, conception and design, Drafting the manuscript. Yanlan Yu: Acquisition of data. Jie Wang, Jing Yan: Analysis and interpretation of data, manuscript revising. Jing Yan, Guoqing Ding: Manuscript revising, final approval of the version to be published, accountable for all aspects of the work.

## CONFLICT OF INTEREST

Jie Wang and Jing Yan were employed by the company Holosensor Medical Ltd. The remaining authors declare that the research was conducted in the absence of any commercial or financial relationships that could be construed as a potential conflict of interest.

## ETHICAL STATEMENT

This research was approved by the Ethics Committee of Sir Run Run Shaw Hospital (#2021‐389‐01) and followed the Declaration of Helsinki. All the individuals recruited in this study were provided with informed consent forms.

## Supporting information


Appendix S1

## Data Availability

All data present in this study are available, but cannot be released to the public due to privacy reasons. However, detailed data can be obtained from the author via email.
