# Supplementary material for: Comprehensive optimization of urinary exfoliated tumor cells tests in bladder cancer with a promising microfluidic platform
Source: Cancer Med. 2022 Dec 25;12(6):7283–93. doi: 10.1002/cam4.5481 (PMC10067033; doi:10.1002/cam4.5481)
Supplement: Supplementary file 1 — Appendix S1 [file CAM4-12-7283-s001.docx]

**Supplement figures and tables:**

**Comprehensive optimization of urinary exfoliated tumor cells tests in bladder cancer with a promising microfluidic platform**

Supplement Tables

**S Table 1**. Cut-off value of pan-CK in UETCs detection.

| **Cut-off** | **Sensitivity** | **Specificity** | **Youden index** |
| --- | --- | --- | --- |
| 32 | 0.742 | 0.385 | 0.127 |
| 33.5 | 0.719 | 0.385 | 0.104 |
| 34.5 | 0.697 | 0.385 | 0.082 |
| 35.5 | 0.685 | 0.385 | 0.07 |
| 38 | 0.674 | 0.462 | 0.136 |
| 40.5 | 0.674 | 0.538 | 0.212 |
| 42 | 0.663 | 0.538 | 0.201 |
| 44 | 0.652 | 0.538 | 0.19 |
| 46 | 0.629 | 0.538 | 0.167 |
| 47.5 | 0.618 | 0.538 | 0.156 |
| 51 | 0.607 | 0.615 | 0.222 |
| 54.5 | 0.584 | 0.615 | 0.199 |
| 57.5 | 0.573 | 0.615 | 0.188 |
| 60.5 | 0.562 | 0.615 | 0.177 |
| 63.5 | 0.562 | 0.692 | 0.254 |
| 68 | 0.539 | 0.692 | 0.231 |
| 73.5 | 0.528 | 0.692 | 0.22 |
| 77.5 | 0.517 | 0.692 | 0.209 |
| 78.5 | 0.483 | 0.692 | 0.175 |
| 79.5 | 0.483 | 0.769 | 0.252 |
| 82.5 | 0.472 | 0.769 | 0.241 |
| 85.5 | 0.461 | 0.769 | 0.23 |
| **86.5** | **0.449** | **0.846** | **0.295** |
| 87.5 | 0.427 | 0.846 | 0.273 |
| 89 | 0.416 | 0.846 | 0.262 |
| 91.5 | 0.393 | 0.846 | 0.239 |
| 94 | 0.382 | 0.846 | 0.228 |
| 95.5 | 0.371 | 0.846 | 0.217 |
| 98 | 0.348 | 0.846 | 0.194 |
| 100.5 | 0.337 | 0.846 | 0.183 |

**S Table 2**. Cut-off value of CK20 in UETCs detection.

| **Cut-off** | **Sensitivity** | **Specificity** | **Youden index** |
| --- | --- | --- | --- |
| 0.5 | 1 | 0.059 | 0 |
| 2 | 1 | 0.118 | 0.059 |
| 3.5 | 1 | 0.353 | 0.118 |
| 5 | 1 | 0.412 | 0.353 |
| 7.5 | 1 | 0.471 | 0.412 |
| 12.5 | 1 | 0.529 | 0.471 |
| 16.5 | 1 | 0.647 | 0.529 |
| 17.5 | 0.917 | 0.824 | 0.647 |
| **19** | **0.917** | **0.882** | **0.741** |
| 22 | 0.833 | 0.882 | 0.799 |
| 26.5 | 0.75 | 0.882 | 0.715 |
| 31.5 | 0.75 | 0.941 | 0.632 |
| 39 | 0.667 | 0.941 | 0.691 |
| 45 | 0.583 | 0.941 | 0.608 |
| 48 | 0.5 | 0.941 | 0.524 |
| 52.5 | 0.417 | 0.941 | 0.441 |

**S Table 3**. Cut-off values of DBC-1 in UETCs detection.

| **Cut-off** | **Sensitivity** | **Specificity** | **Youden index** |
| --- | --- | --- | --- |
| 1 | 1 | 0.167 | 0.167 |
| 2.5 | 1 | 0.25 | 0.25 |
| 3.5 | 1 | 0.333 | 0.333 |
| 5 | 1 | 0.417 | 0.417 |
| 6.5 | 1 | 0.583 | 0.583 |
| 7.5 | 0.938 | 0.583 | 0.521 |
| 9.5 | 0.938 | 0.667 | 0.605 |
| 12 | 0.938 | 0.75 | 0.688 |
| 15 | 0.938 | 0.833 | 0.771 |
| 19.5 | 0.938 | 0.917 | 0.855 |
| **24.5** | **0.938** | **1** | **0.938** |
| 30.5 | 0.875 | 1 | 0.875 |
| 35 | 0.813 | 1 | 0.813 |
| 37 | 0.75 | 1 | 0.75 |
| 41.5 | 0.688 | 1 | 0.688 |
| 49.5 | 0.625 | 1 | 0.625 |
| 54.5 | 0.563 | 1 | 0.563 |
| 63 | 0.438 | 1 | 0.438 |
| 82.5 | 0.375 | 1 | 0.375 |

Supplement figures：


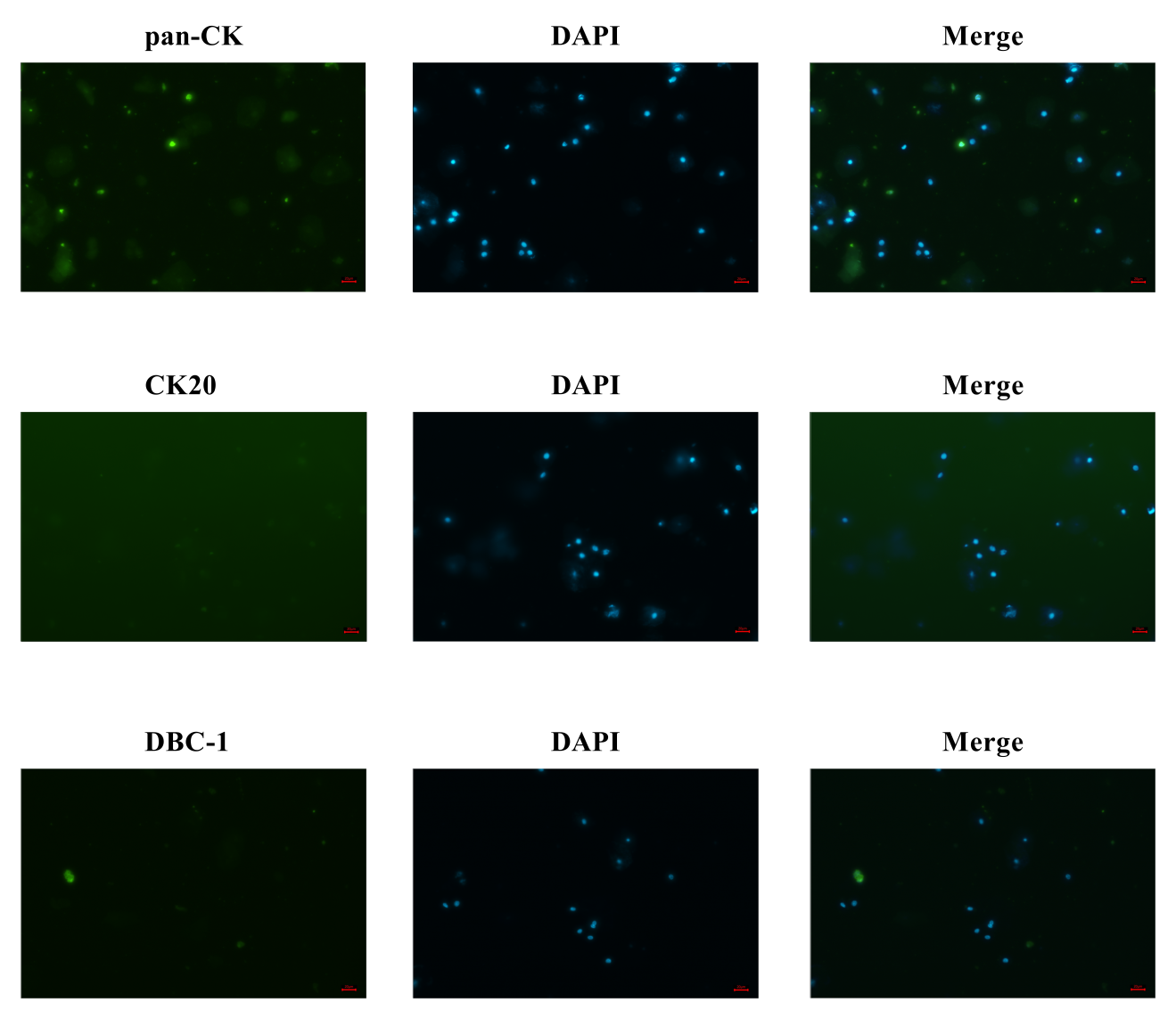


**Supplementary Figure 1: Images of urine cells from healthy individual.** Urine cells of healthy individual identified by pan-CK, CK20 and DBC-1, respectively. Scale bar = 20 μm.


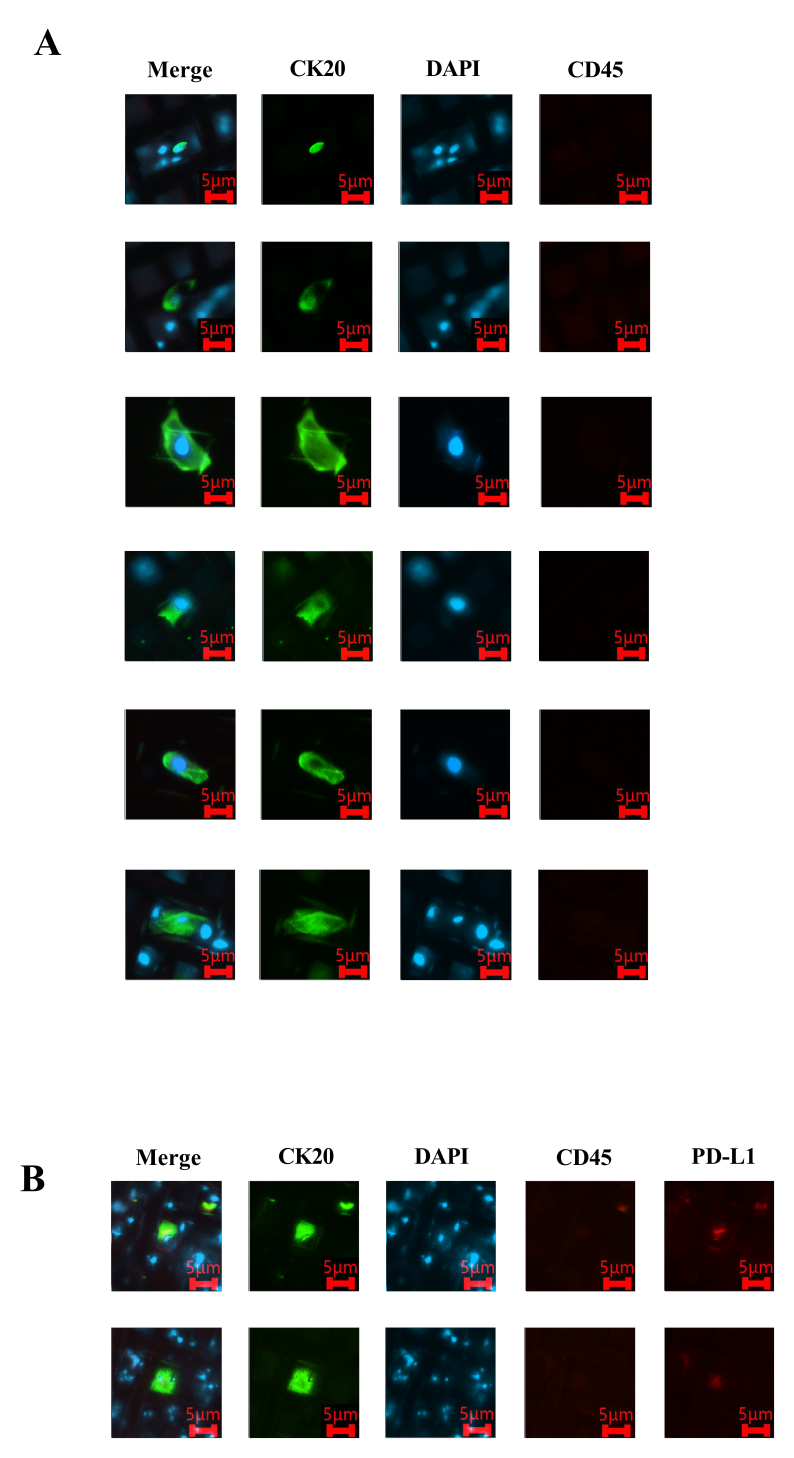


**Supplementary Figure 2: Images of different UETCs in the urine of BLCA patients.** (**A**) Epithelial cells with different morphologies. CK20, nucleus, and CD45 in green, blue and red, respectively. (**B**) PD-L1-positive UETCs. CK20, nucleus, CD45, and PD-L1 in green, blue, red and red, respectively. Scale bar = 5 μm
